# Early-life serotonin dysregulation affects the migration and positioning of cortical interneuron subtypes

**DOI:** 10.1038/tp.2015.147

**Published:** 2015-09-22

**Authors:** S Frazer, K Otomo, A Dayer

**Affiliations:** 1 Department of Mental Health and Psychiatry, University of Geneva Medical School, Geneva, Switzerland; 2 Department of Psychiatry and Basic Neurosciences, University of Geneva Medical School, Geneva, Switzerland

## Abstract

Early-life deficiency of the serotonin transporter (SERT) gives rise to a wide range of psychiatric-relevant phenotypes; however, the molecular and cellular targets of serotonin dyregulation during neural circuit formation remain to be identified. Interestingly, migrating cortical interneurons (INs) derived from the caudal ganglionic eminence (CGE) have been shown to be more responsive to serotonin-mediated signalling compared with INs derived from the medial ganglionic eminence (MGE). Here we investigated the impact of early-life SERT deficiency on the migration and positioning of CGE-derived cortical INs in SERT*-*ko mice and in mice exposed to the SERT inhibitor fluoxetine during the late embryonic period. Using confocal time-lapse imaging and microarray-based expression analysis we found that genetic and pharmacological SERT deficiency significantly increased the migratory speed of CGE-derived INs and affected transcriptional programmes regulating neuronal migration. Postnatal studies revealed that SERT deficiency altered the cortical laminar distribution of subtypes of CGE-derived INs but not MGE-derived INs. More specifically, we found that the distribution of vasointestinal peptide (VIP)-expressing INs in layer 2/3 was abnormal in both genetic and pharmacological SERT-deficiency models. Collectively, these data indicate that early-life SERT deficiency has an impact on the migration and molecular programmes of CGE-derived INs, thus leading to specific alterations in the positioning of VIP-expressing INs. These data add to the growing evidence that early-life serotonin dysregulation affects cortical microcircuit formation and contributes to the emergence of psychiatric-relevant phenotypes.

## Introduction

Vulnerability to psychiatric disorders is likely to be determined by early-life alterations in the formation and plasticity of neural circuits. Cortical microcircuits emerge through sequential cellular events involving the coordinated integration of a wide diversity of neurons in a laminated structure.^1^ Cortical neuron subtypes have different embryonic origins and reach their final cortical laminar position through the process of neuronal migration.^1, 2^ Glutamatergic pyramidal neurons are generated in the ventricular zone of the dorsal pallium and migrate radially towards the pial surface, whereas GABAergic cortical interneurons (INs) are born in a variety of subpallial microdomains and reach the developing cortex through tangential migration.^2, 3^ Alterations in the migration, maturation and function of GABAergic cortical INs have been detected in rodent models of psychiatric disorders and in post-mortem brain tissue from patients with schizophrenia and bipolar disorder.^3, 4, 5, 6^

Among early-life signalling pathways involved in the cortical circuit assembly, the serotonin system has been shown to regulate different types of cellular processes including neuronal migration and thalamocortical wiring.^7, 8, 9^ Serotonin is detected as early as embryonic day (E)10.5 in the mouse forebrain and levels gradually rise during embryonic development.^10^ In rodents, constitutive genetic deletion of the serotonin transporter (SERT) or pharmacological blockade of SERT through exposure to selective serotonin reuptake inhibitors (SSRIs) at prenatal or early postnatal time points gives rise to a variety of persistent behavioural alterations including increased anxiety-like, depressive-like and autistic-like phenotypes.^11, 12, 13, 14^ In humans and non-human primates, a hypofunctional genetic variant in the SERT promoter region (5-HTTLPR, short allele) interacts with early-life adversity to increase risk for affective dysregulation^15, 16, 17, 18^ and modulates emotional circuits.^19, 20^ Finally, prenatal exposure of fetuses to antidepressants such as selective serotonin reuptake inhibitors has been recently shown to increase risk for attention-deficit hyperactivity disorder^21^ and possibly autism spectrum disorders.^22, 23^ Collectively, human and rodent data indicate that SERT deficiency during development constitutes a risk factor for psychiatric-related outcomes. However, the molecular and cell-type-specific mechanisms that link early-life SERT deficiency to psychiatric-relevant phenotypes remain to be understood.

Here we aimed to determine whether early-life SERT deficiency affects the migration and laminar positioning of cortical IN subtypes. Interestingly, a subset of cortical INs (~30%) derived from the caudal ganglionic eminence (CGE) but not from the medial ganglionic eminence (MGE) express the serotonin receptor 3A (5-HT_3A_R).^24, 25, 26^ Recent work from our laboratory has shown that the 5-HT_3A_R specifically controls the embryonic migration and laminar positioning of CGE but not MGE-derived cortical INs.^26^ We thus aimed to determine whether the migration and transcriptional programmes of CGE-derived INs are affected in homozygous SERT-ko mice and in mice exposed to the SERT inhibitor fluoxetine from E14.5 to birth. Using time-lapse imaging and microarray-based gene expression analysis we found that SERT deficiency affected the migratory dynamics of CGE-derived INs as well as a core set of genes involved in regulating neuronal migration. Furthermore, quantification at the postnatal time point indicated that early-life SERT deficiency specifically affected the laminar positioning of CGE-derived INs expressing vasointestinal peptide (VIP) or neuropeptide Y (NPY) but not MGE-derived INs expressing parvalbumin (PV) or somatostatin (SST). Taken together, these data indicate that CGE-derived INs constitute an important cellular target of early-life serotonin dysregulation and that alteration in their migration and positioning could potentially contribute to the complex behavioural deficits induced by early-life SERT deficiency.

## Materials and methods

### Animals

Animal experiments were conducted according to the Swiss and international guidelines and were approved by the local Geneva animal care committee. Adult timed-pregnant mice were obtained by overnight (ON) mating and the following morning was counted as E0.5. Transgenic mice expressing green fluorescent protein (GFP) under the control of the GAD65 regulatory sequences (GAD65-GFP) were used to label CGE-derived cortical INs.^27^ GAD65-GFP mice were maintained on a C57BL/6 background. Timed-pregnant C57BL/6 mice were either obtained from Charles River Laboratories (Saint-Germain-Nuelles, France) or bred in-house. To study genetic SERT deficiency, SERT-ko mice^28^ maintained on a C57BL/6 background were crossed with the GAD65-GFP mice to obtain SERT-ko; GAD65-GFP animals.

### Drug administration

To reduce handling and injection stress, custom-made pellets (Innovative Research of America, Sarasota, FL, USA) were used to deliver fluoxetine continuously for 5 days at a clinically relevant dose (20 mg kg^−1^ per day as previously described^29^). E14.5 GAD65-GFP pregnant dams were implanted subcutaneously with a fluoxetine pellet under brief 1% isoflurane anaesthesia. Control GAD65-GFP mice and SERT-ko; GAD65-GFP mice were implanted with a placebo pellet to control for potential confounding effects of surgery and anaesthesia.

### High-performance liquid chromatography

After giving birth, mice with fluoxetine pellets (*n*=4 from two separate dams) were killed by lethal intraperitoneal injection of pentobarbital (100 mg kg^−1^) and blood from the right atrium was collected in lithium–heparin columns (Microvette, Sarstedt, Nümbrecht, Germany). After centrifugation, the supernatant was stored at −20 °C until further analysis. Brains were extracted and sonicated in ddH_2_O. Concentration of fluoxetine was determined with high-performance liquid chromatography mass spectrometry at the Geneva University Hospital.

### Time-lapse imaging of neuronal migration

Time-lapse imaging of IN migration was performed on E17.5 cortical slices from SERT-ko; GAD65*-*GFP embryos, GAD65-GFP embryos exposed to prenatal fluoxetine (PF) and control GAD65-GFP embryos as previously described.^26^ Time-lapse imaging stacks obtained were piled up using NIS-Elements (Nikon Software, Nikon, Zurich, Switzerland) to obtain orthogonal maximal projections, which were quantified using MetaMorph (Molecular Devices, version 7.7.6, Wokingham, UK). For quantification of cortical invasion, SERT-ko; GAD65-GFP+ INs (*n*=359 cells; *n*=4 slices), PF-exposed GAD65-GFP+ INs (*n*=463 cells; 4=slices) and control GAD65-GFP+ INs (*n*=437 cells; *n*=4 slices) were tracked in an 8-h movie. Slices in each experimental condition were obtained from brains of at least two independent dams. Quantification of speed was performed in three subcompartments: intermediate zone (IZ), cortical plate (CP) and marginal zone (MZ), whose boundaries were manually drawn in MetaMorph based on measurements obtained using Hoechst 33258 (1/10 000, Technologies, Zug, Switzerland) staining. The location of GAD65-GFP+ INs at the start of the time-lapse recording was used to assign migrating INs to one of the three subcompartments. Migration speed was calculated as the total distance travelled by GAD65-GFP+ INs divided by total imaging time excluding the pausing time. The percentage of GAD65-GFP+ INs switching between the CP, IZ and MZ was also calculated.

### Fluorescence-assisted cell sorting and microarrays

Total RNA was collected as previously described.^26^ Briefly, E18.5 brains from SERT-ko; GAD65-GFP, PF-exposed GAD65-GFP mice and control GAD65-GFP mice were dissected in cold Hanks' Balanced Salt Solution (Invitrogen, Life Technologies Europe, Basel, Switzerland). Coronal sections (250 μm) were obtained using a Vibratome (Leica VT100S) placed on porous nitrocellulose membranes (Millicell-CM, Millipore) and cortices containing GAD65-GFP+ were dissected under a Leica M165 FC fluorescent scope. Following dissociation with trypsin, GAD65-GFP+ INs from placebo-pelleted SERT-ko; GAD65-GFP, fluoxetine-pelleted GAD65-GFP mice and control placebo-pelleted GAD65-GFP mice were isolated using fluorescence-assisted cell sorting (FACS) on a FACSVantage in RNAlater solution (Invitrogen). RNA was extracted using an RNeasy Mini kit (Qiagen, Hombrechtikon, Switzerland) in triplicate from three different litters of pooled embryos (*n*=4–5 embryos per FACS) in the three different experimental conditions. For amplification and labelling of total RNA, a small-scale protocol from Affymetrix (High Wycombe, UK) was used as previously described.^26^ Mouse Genome 430 2.0 Arrays (Affymetrix) were scanned on a GC3000 scanner (Affymetrix), and data were analysed using the Partek Genomics suites (version 6.6 beta). The messenger RNA expression levels of genes (ProbeSet, Affymetrix) were compared using a two-way analysis of variance model with uncorrected *P*-values set at <0.05 threshold. Selection of target genes was based on >1.5-fold intensity change in fluoxetine-exposed GAD65-GFP+ INs compared with control GAD65-GFP+ INs, and SERT-ko; GAD65-GFP+ INs compared with control GAD65-GFP+ INs. Selected target genes were validated using quantitative polymerase chain reaction as previously described.^30^ Normalising genes were *Actin-B*, *EEF1*, *RSP9* and *TBP*.

### Tissue processing

SERT-ko; GAD65-GFP, PF-exposed GAD65-GFP and control GAD65-GFP mice were killed by intraperitoneal injection of pentobarbital (100 mg kg^−1^) and were intracardially perfused with 0.9% sodium chloride (NaCl)/Liquemin (2 ml l^−1^), followed by cold 4% paraformaldehyde (PFA)/0.1 m phosphate-buffered saline (PBS) if used for immunohistochemistry (IHC), or 4% PFA/0.1% diethylpyrocarbonate (Sigma, Buchs, Switzerland) if used for *in situ* hybridisation (ISH), After postfixing ON at 4 °C in respective PFA solutions, 50-μm coronal sections were cut on a Vibratome (Leica VT100S) and kept at 4 °C in either 0.1 m PBS if used for IHC, or 0.1 M PBS with 0.1% diethylpyrocarbonate if used for ISH.

### Immunohistochemistry

Antigen retrieval was performed in 10 mM citrate buffer (pH 6.0) at 85 °C for 20 min when required. IHC was performed as previously described^27^ with some changes. Briefly, sections were blocked for 90 min in a blocking buffer (2% Normal Donkey Serum (Gibco, Life Technologies Europe), 0.3% Triton X-100 (Sigma), 0.1% sodium azide (Sigma), 2% bovine serum albumin (Applichem, Baden-Dättwill, Switzerland)) and were incubated with a primary antibody in the blocking buffer for two nights at 4 °C. The primary antibodies used are the following: rat anti-CTIP2 (1:500; Abcam, Cambridge, UK), rabbit anti-CUX1 (1:250; Santa Cruz Biotechnology, Heidelberg, Germany), mouse anti-PV (1:1000; Swant), mouse anti-Reelin (1:500; Abcam), mouse anti-SATB2 (1:500; Abcam), rat anti-SST (1:1000; Chemicon, Millipore) and rabbit anti-VIP (1:500; Abcam). After rinsing, slices were incubated for 2 h at room temperature with the corresponding secondary donkey or goat Alexa-488, -568 and -647 antibodies (Molecular Probes, Invitrogen) raised against the appropriate species at a dilution of 1:1000 and were counterstained with Hoechst 33258 (1:10 000, Life Technologies).

### *In situ* hybridisation

RNA probes were synthesised by *in vitro* transcription. For the SST antisense RNA probe, plasmid template (a kind gift of Paola Arlotta) was linearised with *Eco*R1 (New England Biolabs, MA, USA). After purification, the DNA template was digoxigenin-labelled (Roche, Basel, Switzerland) and polymerised with T3 polymerase (Roche). The NPY antisense RNA probe was synthesised from primers (forward— 5′-CCGGTGGATCTCTTCTCTCA-3′, reverse—5′-CGATGTTAATACGACTCACTATAGGGCAACAACAACAAGGGAAATGG-3′) using the digoxigenin-labelling kit (Roche) and T7 RNA polymerase (Roche). Sections were hybridised as described previously^31^ with some changes. Briefly, sections mounted on Superfrost Plus Ultra slides (Thermo Fisher Scientific, Reinach, Switzerland) were incubated with 2 μg ml^−1^ Proteinase K (EuroBio, Les Ulis, France) followed by inactivation with 2 mg ml^−1^ glycine. Slides were prehybridised in hybridisation buffer (50% deionized formamide, 20% dextran sulphate, 1 mg ml^−1^ salmon sperm (Invitrogen), 1% 100 × Denhart's, 10% 10 × salt, 0.1% diethylpyrocarbonate (Sigma)) for 1 h at 60 °C and then incubated ON with the appropriate probe (2 μl ml^−1^) in hybridisation buffer. After washes, slides were blocked with 10% blocking reagent in 1 × Maleic Acid Buffer/Tween (diluted from 5 × MABT (pH 7.5); 500 mM maleic acid, 750 mM sodium chloride (Sigma-Aldrich) and 0.05% Tween-20), followed by incubation with alkaline phosphatase anti-digoxigenin antibody (1: 2000; Roche) ON at 4 °C. Slices were then washed and incubated ON at 4 °C with 4.5 μl ml^−1^ Nitro blue tetrazolium chloride (Roche) and 3.5 μl ml^−1^ 5-Bromo-4-chloro-3-indolyl-phosphate (Roche) to reveal the staining.

### Quantification of IN laminar positioning

Images were acquired using an epifluorescence microscope (Nikon Eclipse 90i, Nikon) equipped with a × 10 objective (Plan Apo × 10/1, Nikon) or a confocal (Nikon A1R, Nikon) microscope equipped with dry × 10 and × 20 objectives (CFI Plan Apo × 10/0.45 and CFI Plan Apo VC; × 20/0.75, Nikon). Laminar distribution of IN subtypes was quantified on both hemispheres of three coronal sections of brains from SERT-ko, PF-exposed mice and pellet control mice using equally spaced bins apposed at the level of the primary somatosensory cortex. Bins corresponding to cortical layers 1 to 6 were pooled based on cytological differences in the density of Hoechst staining.

### Statistical analysis

No statistics were used to determine optimal group sample size and no randomisation or blinding was used. However, sample sizes were similar to those used in previous publications from our group and others. No samples were excluded from statistical analysis, and normal distribution was assumed for statistical tests. Statistical analyses (GraphPad Prism software, version 6.0, La Jolla, CA, USA) were performed using unpaired two-tailed Student's *t*-test or one-way analysis of variance with Bonferroni's multiple comparisons test.

## Results

### Early-life SERT deficiency increases the migratory speed of CGE-derived INs

**Figure 1:**
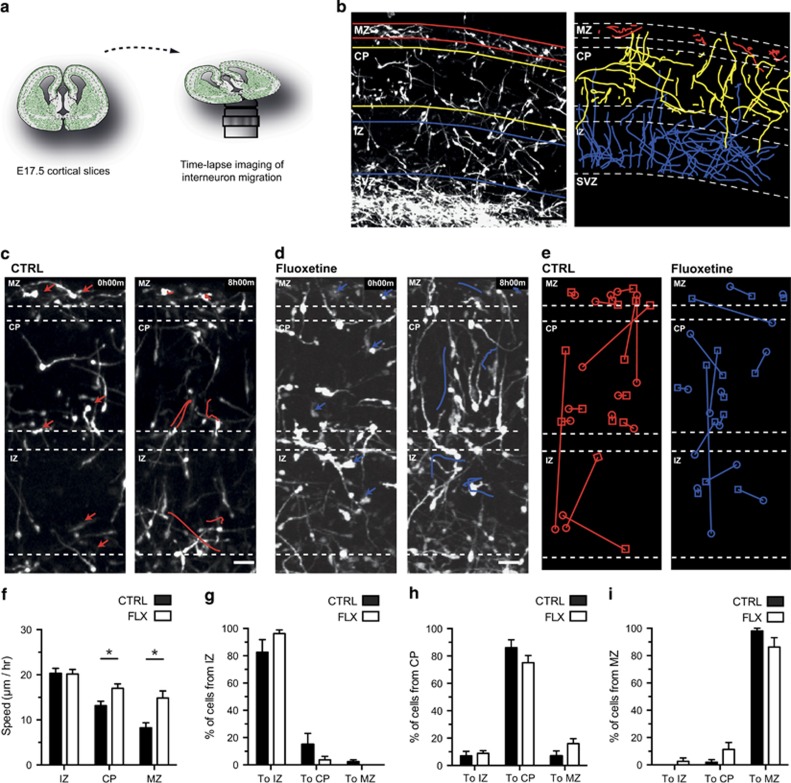
Prenatal fluoxetine exposure increases the migratory speed of caudal ganglionic eminence (CGE)-derived interneurons (INs). (**a**) Migration of CGE-derived GAD65-GFP+ INs was monitored using confocal time-lapse imaging on E17.5 cortical slices for 10–12 h. (**b**) At E17.5, migratory track paths of GAD65-GFP+ INs were analysed in three distinct cortical subcompartments: marginal zone (MZ; red), cortical plate (CP; yellow) and intermediate zone (IZ; blue). (**c, d**) Time-lapse sequence showing illustrative track paths of GAD65-GFP+ INs in the control (red; **c**) and fluoxetine (blue; **d**) condition. Arrows indicate tracked cells. (**e**) GAD65-GFP+ INs switching between the MZ, CP and IZ compartments are illustrated in the control (red) and fluoxetine (blue) conditions. Circles indicate start position of INs and squares end positions of INs. (**f**) Prenatal fluoxetine (FLX) significantly increased the mean migratory speed of GAD65-GFP+ INs in the CP and MZ (*P<0.05, one-way analysis of variance (ANOVA) with Bonferroni's test. *n*=437 cells in CTRL and 463 cells in the FLX condition in at least three independent experiments). (**g–i**) No significant differences were observed between CTRL and FLX in the fraction of cells switching between the IZ, CP and MZ into the different subcompartments. Error bars are means±s.e.m. Scale bars: (**b**) 100 μm, (**c**) 25 μm and (**d**) 25 μm. SVZ, subventricular zone.

To determine the role of early-life SERT deficiency on CGE-derived IN migration, GAD65-GFP+ transgenic mice were used. GAD65-GFP+ cells preferentially label CGE-derived INs and only rarely MGE-derived INs.^26, 27^ To assess the impact of early-life SERT deficiency on CGE-derived IN migration in a clinically relevant paradigm, we first used an *in vivo* pharmacological model of SERT deficiency. Chronic pharmacological blockade of SERT was performed during the late phase of pregnancy by subcutaneously implanting a fluoxetine pellet in E14.5 pregnant dams. Control animals were implanted at E14.5 with a placebo pellet to control for potential confounding effects of brief surgery and anaesthesia. A fluoxetine concentration of 20 mg kg^−1^ per day was chosen on the basis of a previous study indicating that custom-made pellets lead to clinically relevant concentrations of fluoxetine.^29^ High-performance liquid chromatography performed on plasma from dams after delivery confirmed that fluoxetine was in the clinical relevant dosage (172.52±19.93 ng ml^−1^). In brain lysates of P0.5 pups, fluoxetine reached a concentration of 4.25 × 10^3^±561 ng ml^−1^. To determine whether the migration of GAD65-GFP+ INs was modified by SERT pharmacological inhibition, the dynamic migration of GAD65-GFP+ IN<s was monitored during the phase of cortical invasion in E17.5 cortical slices prepared from fluoxetine-exposed and control mice (Figures 1a and b). Confocal time-lapse movies of 8-h duration were obtained and the migratory dynamics of GAD65-GFP+ INs were analysed in the IZ, the CP and the MZ (Figures 1c–e). Quantification revealed that PF exposure significantly increased the velocity of GAD65-GFP+ INs in the MZ and CP but not in the IZ (Figure 1f). No differences were observed in the percentage of GAD65-GFP+ INs switching between the MZ, CP or IZ in the fluoxetine compared with the control condition (Figures 1g–i).

**Figure 2:**
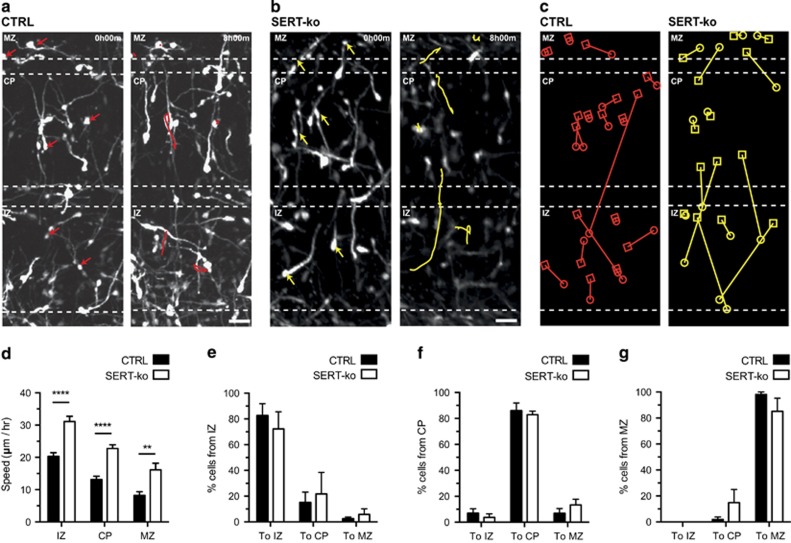
Genetic serotonin transporter (SERT) deletion increases the migratory speed of caudal ganglionic eminence (CGE)-derived interneurons (INs). (**a**, **b**) Time-lapse sequence showing illustrative track paths of control GAD65-GFP+ INs (red; **a**) and SERT-ko; GAD65-GFP+ INs (yellow; **b**) in marginal zone (MZ), cortical plate (CP) and intermediate zone (IZ). Arrows indicate tracked cells. (**c**) GAD65-GFP+ INs switching between the MZ, CP and IZ compartments are illustrated in the control (red) and SERT-ko (yellow) condition. Circles indicate start position of INs and squares end positions of INs. (**d**) The mean migratory speed of GAD65-GFP+ INs is significantly increased in the IZ, CP and MZ in the SERT-ko condition compared with control (****P<0.001, **P<0.01, one-way analysis of variance (ANOVA) with Bonferroni's test. *n*=437 cells in CTRL and 359 cells in SERT-ko condition in at least three independent experiments). (**e–g**) No significant differences were observed between CTRL and SERT-ko in the fraction of cells switching between the IZ, CP or MZ into the different subcompartments. Error bars are means±s.e.m. Scale bars: (**a**) 25 μm and (**b**) 25 μm.

We next determined whether genetic deletion of SERT could affect cortical IN migration. To monitor CGE-derived INs in SERT*-*ko mice, SERT-ko mice were crossed with GAD65-GFP+ mice and the migratory dynamics of SERT-ko; GAD65-GFP+ INs and control GAD65-GFP+ INs were analysed using time-lapse imaging (Figures 2a–c). Quantification revealed that the migratory speed of SERT-ko; GAD65-GFP+ INs was significantly increased in the MZ, CP and IZ compared with control GAD65-GFP+ INs (Figure 2d). No differences were observed in the percentage of GAD65-GFP+ INs switching between the MZ, CP or IZ in SERT-ko; GAD65-GFP compared with control GAD65-GFP+ INs (Figures 2e–g). Taken together, these results indicate that SERT deficiency due to pharmacological blockade or genetic deletion increases the migratory speed of CGE-derived INs during CP invasion.

### Early-life SERT deficiency affects transcriptional programmes involved in neuronal migration

**Figure 3:**
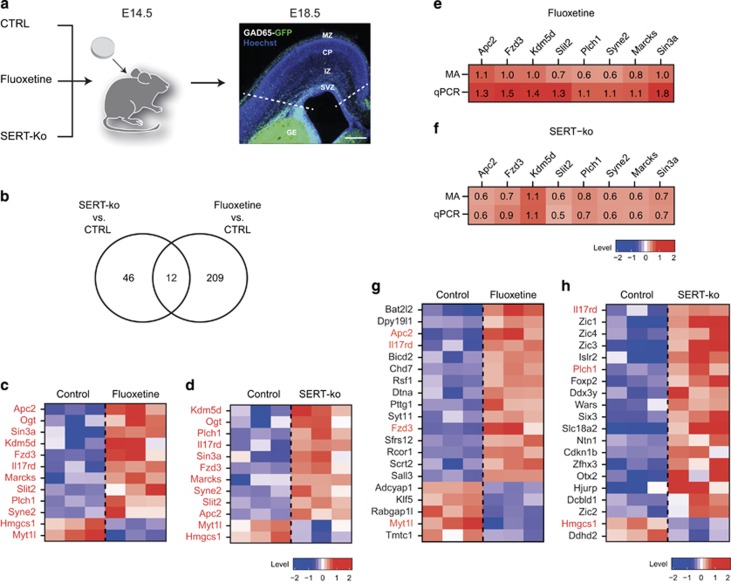
Serotonin transporter (SERT) deficiency alters transcriptional programmes in migrating cortical interneurons (INs). (**a**) Fluoxetine pellets were implanted at E14.5 in GAD65-GFP mice and placebo pellets in GAD65-GFP mice and SERT-ko; GAD65-GFP+ mice. Microdissection of the developing cortex was performed at E18.5 and GAD65-GFP+ INs were isolated using fluorescence-assisted cell sorting. Total RNA was extracted and microarrays were performed (**b**) Venn diagram showing that 12 genes are commonly dysregulated in the fluoxetine and SERT-ko condition compared with control. Genes were identified based on *P*<0.05 (two-way analysis of variance (ANOVA)) and 1.5-fold change (FC) threshold. (**c, d**) Heatmaps showing the 12 commonly dysregulated genes in the control and fluoxetine conditions compared with control. Genes are ranked on the basis of lowest *P*-value scores, and values represent normalised expression levels (log2) in triplicates. (**e, f**) Microarrays (MAs) were validated using quantitative polymerase chain reaction (qPCR) in a selected fraction of commonly dysregulated genes in the fluoxetine (**e**) and SERT-ko condition (**f**). Numbers indicate the mean fold change values (log2). (**g, h**) Heatmaps showing the top 20 genes dysregulated in GAD65-GFP+ INs in fluoxetine (**g**), and SERT-ko (**h**) conditions compared with the control. Genes are ranked on the basis of lowest *P*-value scores and values represent normalised expression levels (log2). Genes dysregulated in both SERT-ko and fluoxetine conditions are in red. Scale bars, (**a**) 200 μm. SVZ, subventricular zone.

To identify transcriptional programmes altered by early-life SERT deficiency, fluoxetine-exposed GAD65-GFP+ INs, SERT-ko; GAD65-GFP+ INs and control GAD65-GFP+INs were isolated from cortical slices at E18.5 using FACS (Figure 3a). Total RNA was extracted and microarray-based gene expression analysis was performed. A total of 221 genes in the fluoxetine condition and 58 genes in the SERT-ko condition displayed a greater than 1.5-fold change in expression levels compared with the control condition (Figure 3b and Supplementary Tables 1–5). A small set of 12 genes was found to be commonly dysregulated in fluoxetine and SERT-ko conditions (Figures 3c and d). Validation of nine selected genes among these twelve genes was performed using quantitative polymerase chain reaction, which confirmed the fold changes observed in the microarray data set (Figure 3e and f). Interestingly, among the commonly dysregulated genes were adenomatosis polyposis coli 2 (*Apc2*),^32, 33^ slit homolog 2 (*Slit2*),^34, 35, 36^ spectrin repeat containing nuclear envelope 2 (*Syne2*)^37^ and frizzled homolog 3 (*Fzd3*),^38, 39^ which have previously been shown to regulate neuronal migration. In addition, among the top 20 genes dysregulated in the fluoxetine-exposed (Figure 3g) or in the SERT-ko condition (Figure 3h), several genes have been shown to regulate neuronal migration including *Scrt2*,^40^ *Dpy19l1*,^41^ *Adcyap1* (ref. 42) and *BicD2* (ref. 43) in the fluoxetine-exposed condition and *Otx2*,^44, 45^ *Foxp-2*,^46^ *Cdkn1B*^47, 48^ and *Ntn-1* (ref. 49) in the SERT-ko condition. Taken together, these results indicate that early-life SERT deficiency alters transcriptional programmes involved in the regulation of neuronal migration.

### The laminar positioning of CGE- but not MGE-derived IN subtypes are specifically affected by early-life SERT deficiency

**Figure 4:**
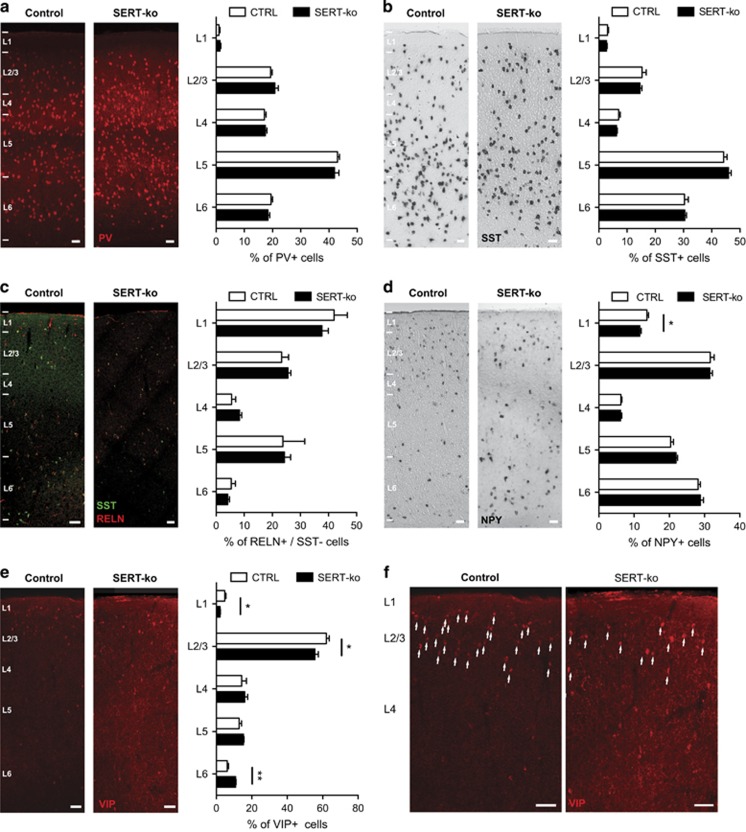
Exposure to prenatal fluoxetine affects the laminar positioning of caudal ganglionic eminence (CGE)-derived but not medial ganglionic eminence (MGE)-derived cortical interneurons (INs). (**a–c**) At P22, no differences were detected in the laminar positioning of MGE-derived PV+ INs (control (CTRL): *n*=16 245 cells in six brains; FLX: *n*=12614 cells in six brains) or SST+ INs (CTRL: *n*=11 072 cells in six brains; FLX: *n*=6398 cells in four brains) as well as CGE-derived RELN+/SST− INs (CTRL: *n*=1421 cells in six brains; FLX: *n*=1358 cells in six brains) in the fluoxetine (FLX) compared with the CTRL condition. (**d**) At P22, a significant difference in the laminar positioning of CGE-derived NPY+ INs was observed in layer 5 in the fluoxetine compared with the control condition (CTRL: *n*=11209 cells in six brains; FLX:*n*=13 003 cells in six brains; *P<0.05 unpaired Student's *t*-test). (**e**) At P22, a significant difference in the laminar positioning of CGE-derived VIP+ INs was observed in layer 2/3 in the fluoxetine compared with the control condition (CTRL: *n*=516 cells in four brains; FLX; *n*=820 cells in five brains; **P<0.01 unpaired Student's *t*-test). Error bars are means±s.e.m. Scale bars, (**a–e**) 50 μm and (**f**) 100 μm. NPY, neuropeptide Y; PV, parvalbumin; RELN, reelin; SST, somatostatin; VIP, vasointestinal peptide.

To determine whether changes in neuronal migration and gene expression induced by early-life SERT deficiency could give rise to alterations in the positioning of cortical IN subtypes, we quantified the distribution of IN subtypes at P22 in the primary somatosensory cortex of fluoxetine-exposed, SERT-ko and control mice. IHC against standard cortical layer markers including CUX1, SATB2 and CTIP2 revealed no striking differences in the laminar organisation of superficial and deep cortical layers in the somatosensory cortex in fluoxetine-exposed or SERT-ko mice compared with control mice (Supplementary Figure 1a). IHC or ISH was next used to analyse the laminar distribution of MGE-derived and CGE-derived cortical INs using IN-specific subclass markers such as PV (IHC) and SST (ISH) to label MGE-derived INs, and reelin (IHC), NPY (ISH) and VIP (IHC) to label CGE-derived INs. Quantification indicated that the laminar distribution of PV+ (Figure 4a) and SST+ (Figure 4b) MGE-derived INs as well as reelin+/SST− CGE-derived IN subtype (Figure 4c) were not altered in the fluoxetine-exposed compared with the control condition. In contrast, the percentage of NPY+ CGE-derived INs was significantly increased in layer 5 (Figure 4d) in the fluoxetine-exposed compared with the control condition. In addition, the percentage of VIP+ CGE-derived INs was significantly decreased in layers 2/3 (Figures 4e and f) in the fluoxetine-exposed compared with the control condition.

**Figure 5:**
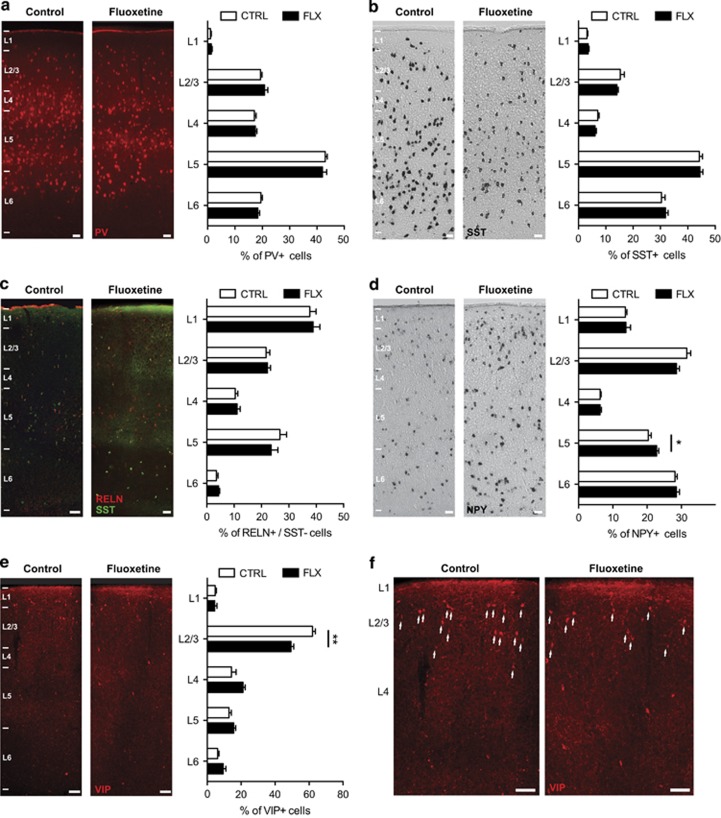
Genetic serotonin transporter (SERT) deletion affects the laminar positioning of caudal ganglionic eminence (CGE)-derived but not medial ganglionic eminence (MGE)-derived cortical interneurons (INs). (**a–c**) At P22, no differences in the laminar positioning of MGE-derived PV+ INs (control (CTRL): *n*=8098 cells in six brains; SERT-ko: *n*=5514 cells in six brains) or SST+ INs (CTRL: *n*=11072 cells in six brains; SERT-ko: *n*=10205 cells in five brains) as well as CGE-derived RELN+/SST− INs (CTRL: *n*=1421 cells in six brains; SERT-ko: *n*=569 cells in four brains) were detected in the SERT-ko compared with the CTRL condition. (**d**) At P22, a significant difference in the laminar positioning of CGE-derived NPY+ INs was observed in layer 1 in the SERT-ko compared with the control condition (CTRL: *n*=11209 cells in six brains; SERT-ko: *n*=9566 cells in five brains; *P<0.05 unpaired Student's *t*-test). (**e**) At P22, a significant difference in the laminar positioning of CGE-derived vasointestinal peptide (VIP)+INs was observed in layers 1, 2/3 and 6 in the SERT-ko compared with the control condition (CTRL: *n*=516 cells in four brains; SERT-ko: *n*=879 cells in five brains; *P<0.05, **P<0.01 unpaired Student's *t*-test). Error bars are means±s.e.m. Scale bars: (**a–e**) 50 μm and (**f**) 100 μm. NPY, neuropeptide Y; PV, parvalbumin; RELN, reelin; SST, somatostatin; VIP, vasointestinal peptide.

Similarly to fluoxetine-exposed mice, no significant differences in the laminar distribution were observed in PV+ (Figure 5a) and SST+ (Figure 5b) MGE-derived INs as well as reelin+/SST− CGE-derived INs (Figure 5c) of SERT-ko mice compared with controls. However, the percentage of NPY+CGE-derived INs was significantly decreased in layer 1 of SERT-ko mice compared with control mice (Figure 5d). In addition, VIP+ CGE-derived INs were found to be significantly modified in layer 1, layers 2/3 and layer 6 of SERT-ko mice compared with control mice (Figure 5e). Collectively, these data indicate that early-life pharmacological or genetic SERT deficiency specifically alters the laminar positioning of VIP+ and NPY+ CGE-derived IN subtypes, but not MGE-derived INs.

## Discussion

Here we investigated the impact of early-life SERT deficiency on the migration, transcriptional programmes and positioning of cortical IN subtypes. We found that early-life SERT deficiency increased the migratory speed of cortical INs during the late phase of cortical invasion and affected transcriptional programmes involved in the regulation of neuronal migration. At later time points, SERT deficiency led to the laminar mispositioning of specific subsets of CGE-derived INs expressing VIP or NPY, but did not modify the positioning of MGE-derived INs. Interestingly, a common mispositioning alteration of VIP+ INs in layer 2/3 was observed in both models of SERT deficiency. Taken together, these data indicate that early-life SERT deficiency preferentially affects the positioning of specific subclasses of cortical INs that are generated in the CGE and not in the MGE.

Cortical INs are composed of a variety of different cell types that have specific roles in regulating information processing in cortical microcircuits.^50^ Developmental studies have revealed that the majority of GABAergic cortical INs (~70%) originate from different microdomains located in the MGE and give rise to fast-spiking PV+ basket cells or SST+ INs.^51, 52, 53^ The remaining fraction of cortical INs (~30%) has been shown to be generated mainly in the CGE^54^ and to a lesser extent in the preoptic area.^55, 56^ CGE-derived INs preferentially target superficial cortical layers and constitute a highly diverse cell population. CGE-derived INs in contrast to MGE-derived INs have been shown to express the 5-HT_3A_R and can be subdivided into reelin+, NPY+ and VIP+ IN subtypes.^24, 25, 26^ Interestingly, *Htr3a* mRNA expression in CGE-derived INs increases about fivefold as they invade the CP.^26^ Functional studies have also demonstrated that the 5-HT_3A_R is required for the proper migration and positioning of CGE but not MGE-derived cortical INs.^26^ Here we found that genetic deletion of SERT and prenatal exposure to fluoxetine increase the migratory speed of CGE-derived INs in cortical slices. These pro-migratory effects could be mediated through the 5-HT_3A_R. Indeed, recent *in vitro* data indicate that serotonin and 5-HT_3A_R activation increase calcium transients as well as the migratory speed of CGE-derived INs isolated during the phase of CP invasion.^26^ Conversely, deletion of the 5-HT_3A_R decreases the migratory speed of CGE-derived INs in cortical slices.^26^ Given that pharmacological blockade or genetic deletion of SERT induces an increase in extracellular levels of serotonin,^57, 58^ we propose that excess of serotonin due to SERT deficiency activates 5-HT_3A_Rs, leading to increases in calcium transients and downstream changes in transcriptional programmes regulating neuronal migration. In addition, serotonin may also have an impact on cortical IN migration by acting through the serotonin receptor 6 (5-HT_6_R),^31^ suggesting that this receptor could also contribute to migratory effects induced by SERT deficiency.

IN migration is a multistep process controlled by a variety of molecular mechanisms such as transcription factors, guidance cues and cytoskeletal regulators.^52^ Using microarrays combined with FACS isolation, we found that genetic and pharmacological SERT deficiency affects transcriptional programmes of migrating CGE-derived INs during the phase of CP invasion. Only a small set of genes was found to be commonly dysregulated in both genetic and pharmacological models of SERT deficiency. Although quantitative polymerase chain reaction confirmed the microarray changes, this was performed on the same complementary DNA library as the microarrays; thus, an independent validation would further strengthen these findings. Interestingly, among dysregulated genes, *Apc2* and *Slit2* have been shown to functionally interact to regulate neuronal migration. *Apc2*, distributed along microtubules and actin fibres, is a key regulator of cytoskeletal dynamics and controls the migration of cortical pyramidal neurons.^32, 33^ SLIT2 is a guidance cue involved in the migration of different types of neurons including subventricular zone progenitors located towards the olfactory bulb,^35^ cholinergic neurons of the basal magnocellular complex^34^ and gonadotropin-releasing hormone neurons.^36^ Interestingly, cerebellar granule cells deficient for *Apc2* are unresponsive to a gradient of SLIT2,^32^ suggesting a functional interplay between these two molecules. Although *Apc2* and *Slit2* do not appear to be involved in the tangential migration of cortical INs,^33, 34^ our microarray data suggest that a functional interaction between *Apc2* and *Slit2* could regulate IN migration at later steps, when INs radially invade the CP. *Syne2, Fzd3* and *Il17rd* were also found to be dysregulated in pharmacological and genetic models of SERT deficiency and could also be interesting candidates to consider in the regulation of CGE-derived IN migration. *Syne2* controls the coupling between the nucleus and the centrosome during radial migration of cortical pyramidal neurons and is essential for cortical lamination.^37^ *Fzd3* is part of the planar cell polarity pathway and has been shown to regulate the migration of facial branchiomotor neurons.^38, 39^ Mutations in *IL17RD* have been associated with Kallmann syndrome,^59^ a human genetic syndrome characterized by alterations in the migration of gonadotropin-releasing hormone and olfactory neurons.^60^ Finally, among other dysregulated genes in both pharmacological and genetic SERT deficiency models, several genes were found to regulate migration in non-neuronal cell types such as Hmg-CoA synthase 1 (*Hmgcs1*) in oligodendrocytes,^61^ O-linked beta-N-acetylglucosamine (*Ogt*) in ovarian cancer cells^62^ and myristoylated alanine-rich C-kinase substrate (*Marcks*) in various different cell types.^63, 64, 65, 66^ Apart from the small set of 12 dysregulated genes in both SERT deficiency models, different sets of genes involved in neuronal migration were found to be specifically dysregulated either in fluoxetine-exposed or SERT-ko conditions. This is not surprising, given that fluoxetine was administered during a restricted developmental time period in contrast to constitutive genetic deletion of SERT. In addition, fluoxetine has been reported to induce off-target transcriptional changes in a genome-wide assay performed in yeast.^67^ SERT-independent effects in biological systems could be because of the fact that fluoxetine directly inhibits several G-coupled protein receptors and ion channels^68, 69, 70^ including TREK channels.^71, 72, 73^ Finally, during development selective serotonin reuptake inhibitors such as citalopram have been shown to regulate thalamic axonal growth in a SERT-independent manner.^74^ These observations thus suggest that some of the fluoxetine-induced transcriptional changes observed in CGE-derived INs could be because of SERT-independent mechanisms. Taken together, our microarray results have led to the identification of transcriptional programmes controlling neuronal migration that are dysregulated in conditions of SERT deficiency. Whether these identified genes are required for the migration of CGE-derived cortical IN remains to be established using cell-type-specific approaches.

Analysis of the laminar positioning of cortical IN subtypes at postnatal time points revealed that early-life SERT deficiency does not affect the laminar distribution of MGE-derived INs, but altered the positioning of specific CGE-derived IN subtypes. In a previous study, we had found that migrating CGE-derived INs are mispositioned at birth in the cortices of SERT-ko mice.^31^ Using subclass markers for CGE-derived INs, we now report that the laminar distribution of a subpopulation of VIP+/5-HT_3A_R-expressing INs was altered in superficial cortical layers 2/3 in both models of early-life SERT deficiency. Interestingly, we found no changes in the positioning CGE-derived reelin+ INs in SERT-deficiency models, whereas reelin+ INs were found to be specifically mispositioned in 5-HT_3A_R-ko mice.^26^ Our data thus suggest that VIP+ INs represent a novel cellular target of early-life SERT deficiency. In the neocortex, VIP+ INs have recently been shown to inhibit MGE-derived SST+ and PV+ INs^75, 76^ and to be strongly recruited by reward and punishment reinforcement signals in the auditory cortex.^75^ It would thus be interesting to determine whether the functional properties of VIP+ INs are affected in SERT-deficiency models and whether CGE-derived IN dysfunction induced by SERT deficiency has a causal role in the psychiatric-relevant phenotypes induced by SERT deficiency. Finally, it should be noted that, in order to isolate GAD65-GFP+ INs from homozygote SERT-ko embryos for the microarray study, it was necessary to mate homozygote SERT-ko; GAD65-GFP+ females to homozygote SERT-ko; GAD65-GFP+ males. Given this strategy, littermate controls were not used in this study. This is a limitation, given that SERT homozygotes and heterozygotes are known to display stress-related behavioural abnormalities that could have an impact on embryonic development.^12^

In conclusion, our study reveals that early-life SERT deficiency affects the migratory dynamics, transcriptional programmes and laminar positioning of a specific subset of cortical INs. The sequential developmental steps that link increased migratory dynamics to the mispositioning of INs in the postnatal cortex remain to be fully understood. In the present model, we propose that early-life SERT deficiency by increasing serotonin extracellular levels will stimulate calcium activity in migrating INs. This will trigger complex transcriptional alterations in a network of migratory and epigenetic pathways, which will ultimately lead to the mispositioning of INs. Interestingly, increased calcium activity during migration has recently been shown to induce mispositioning defects possibly because of the premature differentiation of migrating cortical neurons.^77^ Our data obtained in rodents have potential clinical relevance as antidepressants such as fluoxetine cross the human placenta^78^ to modify fetal physiology^79, 80^ and affect neonatal adaptation,^81, 82, 83, 84, 85^ delay head growth,^86^ may accelerate the closure of critical time periods in speech perception^87^ and increase anxious behaviours in children.^88^ Although the long-term psychiatric-related effects of exposure to antidepressants during pregnancy are difficult to quantify in human cohorts, emerging data suggest that prenatal exposure to antidepressants may increases the risk for autism spectrum disorder^22, 23^ and attention-deficit hyperactivity disorder.^21^ However, it should be noted that these findings need to be further replicated, given the potential confounding effects of maternal depression severity.^89^ Although it is currently very difficult to precisely determine the impact of prenatal selective serotonin reuptake inhibitor exposure on human brain development at a cellular level, our data reveal that, in rodents, early-life SERT deficiency affects specific cellular events involved in cortical circuit formation, which adds to the growing body of evidence that serotonin-related pathways are important regulators of developmental plasticity in health and disease.^8, 9^
