# Supplementary figures and images for: Early-life serotonin dysregulation affects the migration and positioning of cortical interneuron subtypes
Source: Transl Psychiatry. 2015 Sep 22;5(9):e644–. doi: 10.1038/tp.2015.147 (PMC5068808; doi:10.1038/tp.2015.147)

a

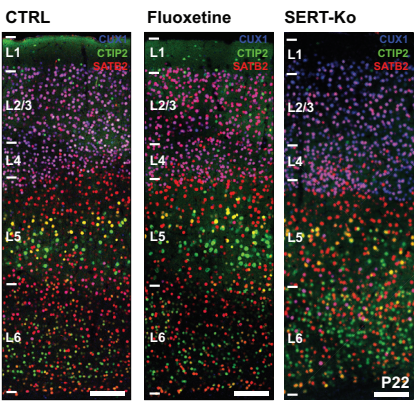

Supplement: Supplementary Figure 1 [file tp2015147x1.pdf]
