# Supplementary material for: Early-life serotonin dysregulation affects the migration and positioning of cortical interneuron subtypes
Source: Transl Psychiatry. 2015 Sep 22;5(9):e644–. doi: 10.1038/tp.2015.147 (PMC5068808; doi:10.1038/tp.2015.147)
Supplement: Supplementary Material [file tp2015147x2.doc]

**Title: Early-life serotonin dysregulation affects the migration and positioning of cortical interneuron subtypes**

Sarah Frazer, Kanako Otomo, Alexandre Dayer

**Supplementary Figure 1.** No major cortical lamination defects are observed in conditions of SERT-deficiency. Immunohistochemistry against CUX1 and SATB2, superficial cortical layer markers, and CTIP2, a deep layer marker, reveals no major changes in the laminar positioning of superficial and deep cortical neurons in fluoxetine-exposed and SERT-ko compared to the control condition. Scale bar: 100 µm.

**Supplementary Table 1.**  Genes dysregulated in *GAD65*-GFP+ interneurons during cortical invasion at E18.5 in fluoxetine and SERT-ko conditions compared to control.

|  | **Gene Symbol** | **Gene Title** | **Fold-Change (KO vs. Ctrl)** | **p-value**  **(KO vs. Ctrl)** | **Fold-Change (FLX vs. Ctrl)** | **p-value**  **(FLX vs. Ctrl)** | **RefSeq Transcript ID** |
| --- | --- | --- | --- | --- | --- | --- | --- |
| 1 | *Il17rd* | interleukin 17 receptor D | 1.68905 | 0.00159414 | 1.8061900 | 0.0008528 | NM_134437 |
| 2 | *Plch1* | phospholipase C, eta 1 | 1.69006 | 0.00412634 | 1.5677200 | 0.0084477 | NM_001177732 /// NM_001177733 /// NM_183191 |
| 3 | *Apc2* | adenomatosis polyposis coli 2 | 1.50247 | 0.0141256 | 2.2001800 | 0.0005694 | NM_011789 |
| 4 | *Fzd3* | frizzled homolog 3 (Drosophila) | 1.61281 | 0.0162202 | 1.9520700 | 0.0035738 | NM_021458 |
| 5 | *Slit2* | slit homolog 2 (Drosophila) | 1.50418 | 0.0210535 | 1.6641600 | 0.0082621 | NM_178804 |
| 6 | *Kdm5d* | lysine (K)-specific demethylase 5D | 2.15177 | 0.0212812 | 1.9530100 | 0.0354525 | NM_011419 |
| 7 | *Sin3a* | transcriptional regulator, SIN3A (yeast) | 1.66765 | 0.0241933 | 1.9968900 | 0.0067347 | NM_001110350 /// NM_001110351 /// NM_011378 |
| 8 | *Marcks* | myristoylated alanine rich protein kinase C substrate | 1.53928 | 0.0282143 | 1.7871800 | 0.0082530 | NM_008538 |
| 9 | *Ogt* | O-linked N-acetylglucosamine (GlcNAc) transferase | 1.78004 | 0.0444037 | 2.0151100 | 0.0216687 | NM_139144 |
| 10 | *Syne2* | synaptic nuclear envelope 2 | 1.53794 | 0.0481647 | 1.5495800 | 0.0454196 | NM_001005510 /// XM_001001846 /// XM_917083 |
| 11 | *Hmgcs1* | 3-hydroxy-3-methylglutaryl-Coenzyme A synthase 1 | -1.56099 | 0.0104511 | -1.6051300 | 0.0079835 | NM_145942 |
| 12 | *Myt1l* | myelin transcription factor 1-like | -1.50634 | 0.0458587 | -2.1146700 | 0.0037325 | NM_001093775 /// NM_001093776 /// NM_001093778 /// NM_008666 /// XM_001480703 |

**Legend:** List of genes displaying a significant up- or down-regulation at E18.5 (> 1.5 fold change, *P* < 0.05) in FACS-isolated GAD65-GFP+ cortical interneurons in fluoxetine (FLX) and SERT-ko (KO) conditions compared to control. Genes are ranked from lowest to highest *P* value scores.

**Supplementary Table 2:** Genes up-regulated in GAD65-GFP+ interneurons during cortical invasion at E18.5 in fluoxetine compared to control condition.

|  | **Gene Symbol** | **Gene Title** | **Fold-Change** | **p-value** | **RefSeq Transcript ID** |
| --- | --- | --- | --- | --- | --- |
| 1 | *Bat2l2* | HLA-B associated transcript 2-like 2 | 2.2212400 | 0.0005391 | NM_001081290 |
| 2 | *Dpy19l1* | dpy-19-like 1 (C. elegans) | 1.5185200 | 0.0005595 | NM_172920 |
| 3 | *Apc2* | adenomatosis polyposis coli 2 | 2.2001800 | 0.0005694 | NM_011789 |
| 4 | *Il17rd* | interleukin 17 receptor D | 1.8061900 | 0.0008528 | NM_134437 |
| 5 | *Bicd2* | bicaudal D homolog 2 (Drosophila) | 1.6477300 | 0.0013286 | NM_001039179 /// NM_001039180 /// NM_029791 |
| 6 | *Chd7* | chromodomain helicase DNA binding protein 7 | 1.5803700 | 0.0014210 | NM_001081417 |
| 7 | *Rsf1* | remodeling and spacing factor 1 | 1.6312300 | 0.0017324 | NM_001081267 |
| 8 | *Dtna* | dystrobrevin alpha | 1.6416400 | 0.0018813 | NM_010087 /// NM_207650 |
| 9 | *Pttg1* | pituitary tumor-transforming gene 1 | 1.6460800 | 0.0025174 | NM_001131054 /// NM_013917 |
| 10 | *Syt11* | synaptotagmin XI | 1.5101400 | 0.0035012 | NM_018804 |
| 11 | *Fzd3* | frizzled homolog 3 (Drosophila) | 1.9520700 | 0.0035738 | NM_021458 |
| 12 | *Sfrs12* | splicing factor, arginine/serine-rich 12 | 1.6462600 | 0.0039257 | NM_172592 |
| 13 | *Rcor1* | REST corepressor 1 | 1.7506100 | 0.0046289 | NM_198023 |
| 14 | *Scrt2* | scratch homolog 2, zinc finger protein (Drosophila) | 1.8277800 | 0.0050286 | NM_001160410 /// XM_619828 /// XM_904795 |
| 15 | *Sall3* | sal-like 3 (Drosophila) | 1.6961500 | 0.0052128 | NM_178280 |
| 16 | *Luc7l3* | LUC7-like 3 (S. cerevisiae) | 1.8528900 | 0.0056185 | NM_026313 |
| 17 | *Creb1* | cAMP responsive element binding protein 1 | 1.6846300 | 0.0057208 | NM_001037726 /// NM_009952 /// NM_133828 |
| 18 | *Scrt2* | scratch homolog 2, zinc finger protein (Drosophila) | 1.5526900 | 0.0057734 | NM_001160410 /// XM_619828 /// XM_904795 |
| 19 | *Prdm16* | PR domain containing 16 | 2.2397000 | 0.0058673 | NM_001177995 /// NM_027504 |
| 20 | *Ilf3* | interleukin enhancer binding factor 3 | 2.0006600 | 0.0064629 | NM_001042707 /// NM_001042708 /// NM_001042709 /// NM_010561 |
| 21 | *Atp1a2* | ATPase, Na+/K+ transporting, alpha 2 polypeptide | 2.1124200 | 0.0066024 | NM_178405 |
| 22 | *Fmn2 /// LOC100044570* | formin 2 /// similar to formin-2 | 1.5993500 | 0.0066302 | NM_019445 /// XR_030793 |
| 23 | *Sin3a* | transcriptional regulator, SIN3A (yeast) | 1.9968900 | 0.0067347 | NM_001110350 /// NM_001110351 /// NM_011378 |
| 24 | *Itga6* | integrin alpha 6 | 1.5408500 | 0.0068609 | NM_008397 |
| 25 | *Slc1a2* | solute carrier family 1 (glial high affinity glutamate transporter), member 2 | 1.6368000 | 0.0074813 | NM_001077514 /// NM_001077515 /// NM_011393 |
| 26 | *Cdk6* | cyclin-dependent kinase 6 | 1.5242600 | 0.0079372 | NM_009873 |
| 27 | *Marcks* | myristoylated alanine rich protein kinase C substrate | 1.7871800 | 0.0082530 | NM_008538 |
| 28 | *Slit2* | slit homolog 2 (Drosophila) | 1.6641600 | 0.0082621 | NM_178804 |
| 29 | *Plch1* | phospholipase C, eta 1 | 1.5677200 | 0.0084477 | NM_001177732 /// NM_001177733 /// NM_183191 |
| 30 | *Cep350* | centrosomal protein 350 | 1.5007700 | 0.0087830 | NM_001039184 /// XM_129509 /// XM_916986 |
| 31 | *Phf3* | PHD finger protein 3 | 1.7756200 | 0.0088350 | NM_001081080 |
| 32 | *Tead1* | TEA domain family member 1 | 1.5629500 | 0.0096715 | NM_001166584 /// NM_001166585 /// NM_009346 |
| 33 | *Phxr4* | per-hexamer repeat gene 4 | 1.6884800 | 0.0104749 | NM_008835 /// NR_028271 |
| 34 | *Sbno1* | sno, strawberry notch homolog 1 (Drosophila) | 1.9064600 | 0.0109718 | NM_001081203 |
| 35 | *Phip* | Pleckstrin homology domain interacting protein | 1.6049200 | 0.0111368 | NM_001081216 |
| 36 | *Ubr2* | ubiquitin protein ligase E3 component n-recognin 2 | 1.5835100 | 0.0114506 | NM_001177374 /// NM_146078 |
| 37 | *Celf1* | CUGBP, Elav-like family member 1 | 1.7159200 | 0.0115727 | NM_017368 /// NM_198683 |
| 38 | *Epb4.1l5* | erythrocyte protein band 4.1-like 5 | 1.5427800 | 0.0120142 | NM_001113416 /// NM_145506 |
| 39 | *Sfrs12* | splicing factor, arginine/serine-rich 12 | 1.5794400 | 0.0124400 | NM_172592 |
| 40 | *Uhrf1* | ubiquitin-like, containing PHD and RING finger domains, 1 | 1.7729800 | 0.0126797 | NM_001111078 /// NM_001111079 /// NM_001111080 /// NM_010931 |
| 41 | *Nipbl* | Nipped-B homolog (Drosophila) | 1.5167500 | 0.0128384 | NM_027707 /// NM_201232 |
| 42 | *Mki67* | antigen identified by monoclonal antibody Ki 67 | 1.7985600 | 0.0130700 | NM_001081117 /// XM_001000692 /// XM_001479849 |
| 43 | *Zfp292* | zinc finger protein 292 | 1.5037900 | 0.0137321 | NM_013889 /// XM_620009 /// XM_909747 |
| 44 | *Chtf18* | CTF18, chromosome transmission fidelity factor 18 homolog (S. cerevisiae) | 1.5121900 | 0.0137589 | NM_145409 |
| 45 | *Zbtb20* | zinc finger and BTB domain containing 20 | 1.9482300 | 0.0140714 | NM_019778 /// NM_181058 |
| 46 | *Cep164* | centrosomal protein 164 | 1.7046800 | 0.0142012 | NM_001081373 |
| 47 | *Myo10* | myosin X | 1.9300100 | 0.0145124 | NM_019472 |
| 48 | *Tcf7l2* | transcription factor 7-like 2, T-cell specific, HMG-box | 2.9912200 | 0.0147444 | NM_001142918 /// NM_001142919 /// NM_001142920 /// NM_001142921 /// NM_001142922 |
| 49 | *Ptpn11* | protein tyrosine phosphatase, non-receptor type 11 | 1.7043100 | 0.0150259 | NM_001109992 /// NM_011202 |
| 50 | *Dnmt3a* | DNA methyltransferase 3A | 1.5065400 | 0.0153913 | NM_007872 /// NM_153743 |
| 51 | *Sept10* | septin 10 | 1.5439400 | 0.0153933 | NM_001024910 /// NM_001024911 |
| 52 | *Fryl* | furry homolog-like (Drosophila) | 1.6082900 | 0.0154538 | NM_028194 /// NM_177136 |
| 53 | *Cxxc5* | CXXC finger 5 | 1.6078300 | 0.0154618 | NM_133687 |
| 54 | *Col4a4* | collagen, type IV, alpha 4 | 1.5949700 | 0.0164379 | NM_007735 |
| 55 | *Cytsa* | cytospin A | 1.6741000 | 0.0164828 | NM_001145826 /// NM_153406 |
| 56 | *Bcan* | brevican | 1.5803300 | 0.0173846 | NM_001109758 /// NM_007529 |
| 57 | *Eomes* | eomesodermin homolog (Xenopus laevis) | 1.6477400 | 0.0175244 | NM_001164789 /// NM_010136 |
| 58 | *Kif1b* | kinesin family member 1B | 1.7914700 | 0.0176068 | NM_008441 /// NM_207682 |
| 59 | *Cul5* | cullin 5 | 1.7700100 | 0.0177433 | NM_001161618 /// NM_027807 |
| 60 | *Ankrd11* | ankyrin repeat domain 11 | 1.7685300 | 0.0180869 | NM_001081379 |
| 61 | *Agap1* | ArfGAP with GTPase domain, ankyrin repeat and PH domain 1 | 1.5143200 | 0.0192688 | NM_001037136 /// NM_178119 |
| 62 | *LOC100046032 /// Pou3f2* | similar to brain-2 class III POU-domain protein /// POU domain, class 3, transcr | 2.2698700 | 0.0197336 | NM_008899 /// XM_001475430 |
| 63 | *Id4 /// LOC100045546* | inhibitor of DNA binding 4 /// similar to Id4 | 1.6293500 | 0.0197562 | NM_031166 /// XM_001474498 |
| 64 | *Cpsf6* | cleavage and polyadenylation specific factor 6 | 2.1074300 | 0.0200229 | NM_001013391 |
| 65 | *Unc5d* | unc-5 homolog D (C. elegans) | 1.5133400 | 0.0201973 | NM_153135 |
| 66 | *Sh3rf1* | SH3 domain containing ring finger 1 | 1.7128000 | 0.0202813 | NM_021506 |
| 67 | *Ascl1* | achaete-scute complex homolog 1 (Drosophila) | 1.5858700 | 0.0203059 | NM_008553 |
| 68 | *Eif4e3* | eukaryotic translation initiation factor 4E member 3 | 1.5770400 | 0.0204119 | NM_025829 |
| 69 | *Prdm16* | PR domain containing 16 | 1.5470500 | 0.0214517 | NM_001177995 /// NM_027504 |
| 70 | *Ogt* | O-linked N-acetylglucosamine (GlcNAc) transferase | 2.0151100 | 0.0216687 | NM_139144 |
| 71 | *Rc3h2* | ring finger and CCCH-type zinc finger domains 2 | 2.0002200 | 0.0217266 | NM_001100591 /// XM_130233 /// XM_925271 |
| 72 | *Ncor2* | nuclear receptor co-repressor 2 | 1.5133900 | 0.0222693 | NM_011424 |
| 73 | *Rapgef6* | Rap guanine nucleotide exchange factor (GEF) 6 | 1.5976900 | 0.0226398 | NM_175258 |
| 74 | *Ltbp3* | latent transforming growth factor beta binding protein 3 | 1.5140100 | 0.0237844 | NM_008520 |
| 75 | *Sfrs15* | splicing factor, arginine/serine-rich 15 | 2.0813700 | 0.0245640 | NM_178923 |
| 76 | *Slc1a3* | solute carrier family 1 (glial high affinity glutamate transporter), member 3 | 1.6168300 | 0.0249096 | NM_148938 |
| 77 | *Tcof1* | Treacher Collins Franceschetti syndrome 1, homolog | 1.6629700 | 0.0251364 | NM_011552 |
| 78 | *Fam53b* | family with sequence similarity 53, member B | 1.6676600 | 0.0252072 | NM_175268 /// NM_212473 |
| 79 | *Ggps1* | geranylgeranyl diphosphate synthase 1 | 1.5590900 | 0.0252635 | NM_010282 |
| 80 | *Trrap* | transformation/transcription domain-associated protein | 1.7249100 | 0.0261724 | NM_001081362 |
| 81 | *Ankhd1 /// Eif4ebp3* | ankyrin repeat and KH domain containing 1 /// eukaryotic translation initiation | 1.5867500 | 0.0271220 | NM_175375 /// NM_201256 /// XM_892728 /// XM_900248 /// XM_900256 /// XM_900265 |
| 82 | *Gfap* | glial fibrillary acidic protein | 1.8602400 | 0.0277711 | NM_001131020 /// NM_010277 |
| 83 | *Nek1* | NIMA (never in mitosis gene a)-related expressed kinase 1 | 2.3244300 | 0.0296468 | NM_175089 |
| 84 | *Sorl1* | sortilin-related receptor, LDLR class A repeats-containing | 1.5034400 | 0.0305561 | NM_011436 |
| 85 | *March7* | membrane-associated ring finger (C3HC4) 7 | 2.0447400 | 0.0312311 | NM_020575 |
| 86 | *Whsc1l1* | Wolf-Hirschhorn syndrome candidate 1-like 1 (human) | 1.6321500 | 0.0313864 | NM_001001735 /// NM_001081269 |
| 87 | *Smc2* | structural maintenance of chromosomes 2 | 1.6812800 | 0.0323745 | NM_008017 |
| 88 | *Fzd2* | frizzled homolog 2 (Drosophila) | 1.6042300 | 0.0337356 | NM_020510 |
| 89 | *Golga4* | golgi autoantigen, golgin subfamily a, 4 | 1.5415600 | 0.0338645 | NM_018748 |
| 90 | *Atp1a2* | ATPase, Na+/K+ transporting, alpha 2 polypeptide | 1.6500500 | 0.0339072 | NM_178405 |
| 91 | *Sbf2* | SET binding factor 2 | 1.5752300 | 0.0343430 | NM_177324 /// XM_001477215 /// XM_001478567 |
| 92 | *Zfp292* | zinc finger protein 292 | 1.7096000 | 0.0346625 | NM_013889 /// XM_620009 /// XM_909747 |
| 93 | *H2-D1* | histocompatibility 2, D region locus 1 | 1.5394800 | 0.0346771 | NM_010380 |
| 94 | *Kdm5d* | lysine (K)-specific demethylase 5D | 1.9530100 | 0.0354525 | NM_011419 |
| 95 | *Bicd2* | bicaudal D homolog 2 (Drosophila) | 1.5807100 | 0.0355935 | NM_001039179 /// NM_001039180 /// NM_029791 |
| 96 | *Ptpn21* | protein tyrosine phosphatase, non-receptor type 21 | 1.5741100 | 0.0374956 | NM_001146199 /// NM_011877 |
| 97 | *Pum1* | pumilio 1 (Drosophila) | 1.7358500 | 0.0375056 | NM_001159603 /// NM_001159604 /// NM_001159605 /// NM_001159606 /// NM_030722 |
| 98 | *Sema5a* | sema domain, seven thrombospondin repeats (type 1 and type 1-like), transmembrane | 2.0720900 | 0.0381951 | NM_009154 |
| 99 | *Smc2* | Structural maintenance of chromosomes 2 | 1.5245900 | 0.0382237 | NM_008017 |
| 100 | *Naa16* | N(alpha)-acetyltransferase 16, NatA auxiliary subunit | 1.7163400 | 0.0383263 | NM_025832 |
| 101 | *Myt1* | myelin transcription factor 1 | 2.9052800 | 0.0391027 | NM_001171615 /// NM_001171616 /// NM_001171680 /// NM_008665 |
| 102 | *Upf1* | UPF1 regulator of nonsense transcripts homolog (yeast) | 1.5147600 | 0.0400944 | NM_001122829 /// NM_030680 |
| 103 | *Bcl11a* | B-cell CLL/lymphoma 11A (zinc finger protein) | 2.0830500 | 0.0404029 | NM_001159289 /// NM_001159290 /// NM_016707 |
| 104 | *Vat1* | vesicle amine transport protein 1 homolog (T californica) | 1.5738900 | 0.0408893 | NM_012037 |
| 105 | *Pard3* | par-3 (partitioning defective 3) homolog (C. elegans) | 1.8104800 | 0.0412654 | NM_001013580 /// NM_001013581 /// NM_001122850 /// NM_033620 |
| 106 | *Col11a1* | collagen, type XI, alpha 1 | 1.5901600 | 0.0412951 | NM_007729 |
| 107 | *Rbms2* | RNA binding motif, single stranded interacting protein 2 | 1.5343600 | 0.0417879 | NM_001039080 /// NM_019711 |
| 108 | *Celsr2* | cadherin, EGF LAG seven-pass G-type receptor 2 (flamingo homolog, Drosophila) | 1.6195100 | 0.0421746 | NM_001004177 /// NM_017392 |
| 109 | *Mll1* | myeloid/lymphoid or mixed-lineage leukemia 1 | 1.5019000 | 0.0423063 | NM_001081049 |
| 110 | *Eya1* | eyes absent 1 homolog (Drosophila) | 1.5990600 | 0.0443487 | NM_010164 |
| 111 | *Baz2b* | bromodomain adjacent to zinc finger domain, 2B | 1.6374200 | 0.0444467 | NM_001001182 |
| 112 | *Syne2* | synaptic nuclear envelope 2 | 1.5495800 | 0.0454196 | NM_001005510 /// XM_001001846 /// XM_917083 |
| 113 | *Rbm26* | RNA binding motif protein 26 | 1.7309300 | 0.0458229 | NM_134077 |
| 114 | *Cald1* | caldesmon 1 | 1.5226500 | 0.0458525 | NM_145575 |
| 115 | *Rbmx* | RNA binding motif protein, X chromosome | 1.5777600 | 0.0467244 | NM_001166623 /// NM_011252 /// NR_029425 |
| 116 | *Smg6* | Smg-6 homolog, nonsense mediated mRNA decay factor (C. elegans) | 1.7420900 | 0.0469017 | NM_001002764 |
| 117 | *Apc* | adenomatosis polyposis coli | 1.6353900 | 0.0474629 | NM_007462 |
| 118 | *Zfc3h1* | zinc finger, C3H1-type containing | 1.5604400 | 0.0474921 | NM_001033261 |

**Legend:** List of genes displaying a significant up-regulation at E18.5 (> 1.5 fold increase, *P* < 0.05) in FACS-isolated *GAD65*-GFP+ cortical interneurons in fluoxetine compared to control condition. Genes are ranked from lowest to highest *P* value scores. Genes labelled in red correspond to genes also significantly dysregulated in the SERT-ko condition.

**Supplementary Table 3:** Genes down-regulated in *GAD65*-GFP+ interneurons during cortical invasion at E18.5 in fluoxetine compared to control condition.

|  | **Gene Symbol** | **Gene Title** | **Fold-Change** | **p-value** | **RefSeq Transcript ID** |
| --- | --- | --- | --- | --- | --- |
| 1 | *Adcyap1* | adenylate cyclase activating polypeptide 1 | -1.5792400 | 0.0002048 | NM_009625 |
| 2 | *Klf5* | Kruppel-like factor 5 | -1.6155900 | 0.0012992 | NM_009769 |
| 3 | *Rabgap1l* | RAB GTPase activating protein 1-like | -1.8984800 | 0.0026364 | NM_001038621 /// NM_013862 |
| 4 | *Myt1l* | myelin transcription factor 1-like | -2.1146700 | 0.0037325 | NM_001093775 /// NM_001093776 /// NM_001093778 /// NM_008666 /// XM_001480703 |
| 5 | *Tmtc1* | transmembrane and tetratricopeptide repeat containing 1 | -1.5370600 | 0.0051507 | NM_198967 |
| 6 | *Zscan18* | zinc finger and SCAN domain containing 18 | -1.8259000 | 0.0060062 | NM_001017955 /// XM_001476882 /// XM_001478350 |
| 7 | *Hmgcs1* | 3-hydroxy-3-methylglutaryl-Coenzyme A synthase 1 | -1.6051300 | 0.0079835 | NM_145942 |
| 8 | *Prkar2b* | protein kinase, cAMP dependent regulatory, type II beta | -1.6036400 | 0.0089272 | NM_011158 |
| 9 | *Mtap1b* | microtubule-associated protein 1B | -2.3988100 | 0.0089411 | NM_008634 |
| 10 | *Dlg2* | discs, large homolog 2 (Drosophila) | -1.7012000 | 0.0090038 | NM_011807 |
| 11 | *H2-T24* | histocompatibility 2, T region locus 24 | -1.5056100 | 0.0096245 | NM_008207 |
| 12 | *Scd1* | stearoyl-Coenzyme A desaturase 1 | -1.5214500 | 0.0110680 | NM_009127 |
| 13 | *Zdhhc20* | zinc finger, DHHC domain containing 20 | -1.5165500 | 0.0114383 | NM_029492 |
| 14 | *Prrg3* | proline rich Gla (G-carboxyglutamic acid) 3 (transmembrane) | -1.5883500 | 0.0114592 | NM_001081135 |
| 15 | *Slc16a7* | solute carrier family 16 (monocarboxylic acid transporters), member 7 | -2.0748900 | 0.0118179 | NM_011391 |
| 16 | *Dpp10 /// LOC100047231* | dipeptidylpeptidase 10 /// similar to Dipeptidylpeptidase 10 | -2.2217600 | 0.0126822 | NM_199021 /// XR_033391 |
| 17 | *Slco4a1* | solute carrier organic anion transporter family, member 4a1 | -1.8235600 | 0.0147173 | NM_148933 |
| 18 | *Gria4* | glutamate receptor, ionotropic, AMPA4 (alpha 4) | -1.7350300 | 0.0152707 | NM_001113180 /// NM_001113181 /// NM_019691 |
| 19 | *Tbl1xr1* | transducin (beta)-like 1X-linked receptor 1 | -1.7081900 | 0.0155732 | NM_030732 |
| 20 | *Fbll1* | fibrillarin-like 1 | -1.5342400 | 0.0159375 | NM_001004147 |
| 21 | *Ydjc* | YdjC homolog (bacterial) | -1.6464300 | 0.0161038 | NM_026940 |
| 22 | *Prkar2b* | protein kinase, cAMP dependent regulatory, type II beta | -1.7167800 | 0.0165234 | NM_011158 |
| 23 | *Gria4* | glutamate receptor, ionotropic, AMPA4 (alpha 4) | -1.5885100 | 0.0167776 | NM_001113180 /// NM_001113181 /// NM_019691 |
| 24 | *Trub1* | TruB pseudouridine (psi) synthase homolog 1 (E. coli) | -1.8200100 | 0.0175685 | NM_028115 /// NM_029839 |
| 25 | *Mcts1* | malignant T cell amplified sequence 1 | -1.6276100 | 0.0196004 | NM_026902 |
| 26 | *D3Bwg0562e* | DNA segment, Chr 3, Brigham & Women's Genetics 0562 expressed | -1.5766500 | 0.0197414 | NM_177664 |
| 27 | *Fhl2* | four and a half LIM domains 2 | -1.7213900 | 0.0205549 | NM_010212 |
| 28 | *Ipcef1* | interaction protein for cytohesin exchange factors 1 | -1.5460100 | 0.0208204 | NM_001033391 /// NM_001170800 /// NM_001170801 /// NM_001170802 |
| 29 | *Sphkap* | SPHK1 interactor, AKAP domain containing | -1.8758900 | 0.0208867 | NM_172430 |
| 30 | *Nefm* | neurofilament, medium polypeptide | -2.2441900 | 0.0219809 | NM_008691 |
| 31 | *Id2* | inhibitor of DNA binding 2 | -1.5288400 | 0.0234818 | NM_010496 |
| 32 | *Homer1* | homer homolog 1 (Drosophila) | -1.5196600 | 0.0248148 | NM_011982 /// NM_147176 /// NM_152134 |
| 33 | *Nr4a2* | nuclear receptor subfamily 4, group A, member 2 | -2.0593500 | 0.0252845 | NM_001139509 /// NM_013613 |
| 34 | *Asns* | asparagine synthetase | -1.5969000 | 0.0255837 | NM_012055 |
| 35 | *Scg2* | secretogranin II | -3.2708500 | 0.0262791 | NM_009129 |
| 36 | *Syt1* | synaptotagmin I | -2.2940300 | 0.0264176 | NM_009306 |
| 37 | *Kcnq5* | potassium voltage-gated channel, subfamily Q, member 5 | -1.8590100 | 0.0271579 | NM_001160139 /// NM_023872 |
| 38 | *Slc7a14* | solute carrier family 7 (cationic amino acid transporter, y+ system), member 14 | -1.5569600 | 0.0276690 | NM_172861 |
| 39 | *Arl6ip5* | ADP-ribosylation factor-like 6 interacting protein 5 | -1.5867200 | 0.0281836 | NM_022992 |
| 40 | *Ptpro* | protein tyrosine phosphatase, receptor type, O | -1.9048700 | 0.0288600 | NM_001164401 /// NM_001164402 /// NM_001164403 /// NM_011216 |
| 41 | *Cdkl2* | cyclin-dependent kinase-like 2 (CDC2-related kinase) | -1.8884000 | 0.0290213 | NM_016912 /// NM_177270 |
| 42 | *Setd5* | SET domain containing 5 | -1.6106500 | 0.0290358 | NM_028385 |
| 43 | *Ccbe1* | collagen and calcium binding EGF domains 1 | -2.6139300 | 0.0291676 | NM_178793 |
| 44 | *Dync1i1* | dynein cytoplasmic 1 intermediate chain 1 | -2.3324000 | 0.0292176 | NM_010063 |
| 45 | *Kcnu1* | potassium channel, subfamily U, member 1 | -1.5734100 | 0.0292582 | NM_008432 |
| 46 | *Gda* | guanine deaminase | -1.6986200 | 0.0292921 | NM_010266 |
| 47 | *Plekha1* | pleckstrin homology domain containing, family A | -1.5428000 | 0.0302237 | NM_133942 |
| 48 | *Tnrc6c* | trinucleotide repeat containing 6C | -1.6836800 | 0.0302472 | NM_198022 |
| 49 | *Rabgap1l* | RAB GTPase activating protein 1-like | -1.5310400 | 0.0305523 | NM_001038621 /// NM_013862 |
| 50 | *Gpr135* | G protein-coupled receptor 135 | -1.9407700 | 0.0312423 | NM_181752 |
| 51 | *Nxph2* | neurexophilin 2 | -2.7033500 | 0.0313180 | NM_008752 |
| 52 | *Ccbe1* | collagen and calcium binding EGF domains 1 | -3.1084900 | 0.0313971 | NM_178793 |
| 53 | *Frmd3* | FERM domain containing 3 | -1.6014600 | 0.0320702 | NM_001163732 /// NM_172869 |
| 54 | *Gpr135* | G protein-coupled receptor 135 | -1.7176600 | 0.0322927 | NM_181752 |
| 55 | *Spin4* | spindlin family, member 4 | -1.6858300 | 0.0323100 | NM_178753 |
| 56 | *Rem2* | rad and gem related GTP binding protein 2 | -2.0351500 | 0.0324251 | NM_080726 |
| 57 | *Rnd1* | Rho family GTPase 1 | -1.5005300 | 0.0334046 | NM_172612 |
| 58 | *Rps4y2* | ribosomal protein S4, Y-linked 2 | -1.7746300 | 0.0335604 | NR_003634 /// XR_035148 /// XR_035151 |
| 59 | *Dab1* | disabled homolog 1 (Drosophila) | -1.7901500 | 0.0343557 | NM_010014 /// NM_177259 |
| 60 | *Uchl5* | Ubiquitin carboxyl-terminal esterase L5 | -1.6050800 | 0.0358791 | NM_001159866 /// NM_019562 |
| 61 | *Paqr9* | progestin and adipoQ receptor family member IX | -1.6663500 | 0.0361542 | NM_198414 |
| 62 | *Pcsk2* | proprotein convertase subtilisin/kexin type 2 | -1.6761800 | 0.0363504 | NM_008792 |
| 63 | *Fam118a* | family with sequence similarity 118, member A | -1.6385700 | 0.0366143 | NM_133750 /// NM_177067 |
| 64 | *Ndrg1* | N-myc downstream regulated gene 1 | -2.3357500 | 0.0367945 | NM_008681 |
| 65 | *Sec61a2* | Sec61, alpha subunit 2 (S. cerevisiae) | -1.5468100 | 0.0369508 | NM_021305 |
| 66 | *Mtus2* | microtubule associated tumor suppressor candidate 2 | -1.5434100 | 0.0372712 | NM_029920 |
| 67 | *Ddb2* | damage specific DNA binding protein 2 | -1.5885700 | 0.0375955 | NM_028119 |
| 68 | *Cacnb4* | calcium channel, voltage-dependent, beta 4 subunit | -1.9901500 | 0.0377162 | NM_001037099 /// NM_146123 |
| 69 | *Slc16a14* | solute carrier family 16 (monocarboxylic acid transporters), member 14 | -2.1676600 | 0.0378259 | NM_027921 |
| 70 | *Fam19a2* | family with sequence similarity 19, member A2 | -2.3967800 | 0.0387967 | NM_182807 |
| 71 | *Gpr62* | G protein-coupled receptor 62 | -1.7386500 | 0.0393816 | NM_001159652 /// XM_975079 |
| 72 | *Ssbp2* | single-stranded DNA binding protein 2 | -2.0959000 | 0.0395170 | NM_024186 /// NM_024272 |
| 73 | *Gabra1* | gamma-aminobutyric acid (GABA) A receptor, subunit alpha 1 | -2.8675200 | 0.0404782 | NM_010250 |
| 74 | *Amy1* | amylase 1, salivary | -1.7879900 | 0.0405553 | NM_001110505 /// NM_007446 |
| 75 | *Mapk10* | mitogen-activated protein kinase 10 | -1.6924600 | 0.0408388 | NM_001081567 /// NM_009158 |
| 76 | *Rps6ka5* | ribosomal protein S6 kinase, polypeptide 5 | -1.5062800 | 0.0409478 | NM_153587 |
| 77 | *Arg2* | arginase type II | -1.5184900 | 0.0409812 | NM_009705 |
| 78 | *Mef2c* | myocyte enhancer factor 2C | -1.7748600 | 0.0418927 | NM_001170537 /// NM_025282 |
| 79 | *Scn3b* | sodium channel, voltage-gated, type III, beta | -1.5945900 | 0.0432851 | NM_001083917 /// NM_153522 /// NM_178227 |
| 80 | *Csrnp3* | cysteine-serine-rich nuclear protein 3 | -1.9005800 | 0.0433183 | NM_153409 /// NM_178634 |
| 81 | *Sla* | src-like adaptor | -3.1779000 | 0.0434144 | NM_001029841 /// NM_009192 |
| 82 | *Dact1* | dapper homolog 1, antagonist of beta-catenin (xenopus) | -1.8176400 | 0.0440218 | NM_021532 |
| 83 | *Sst* | somatostatin | -3.1718900 | 0.0446801 | NM_009215 |
| 84 | *Kcnf1* | potassium voltage-gated channel, subfamily F, member 1 | -2.0101800 | 0.0447206 | NM_201531 |
| 85 | *Rufy2* | RUN and FYVE domain-containing 2 | -1.6805600 | 0.0456580 | NM_027425 |
| 86 | *Met* | met proto-oncogene | -1.6986800 | 0.0456757 | NM_008591 |
| 87 | *Nin* | ninein | -1.8849100 | 0.0457827 | NM_001081453 /// NM_008697 |
| 88 | *Slc6a15* | solute carrier family 6 (neurotransmitter transporter), member 15 | -1.8080300 | 0.0459923 | NM_175328 /// XR_035425 /// XR_035434 |
| 89 | *Clca1 /// Clca2* | chloride channel calcium activated 1 /// chloride channel calcium activated 2 | -2.6467300 | 0.0460230 | NM_009899 /// NM_030601 |
| 90 | *Dnaja4* | DnaJ (Hsp40) homolog, subfamily A, member 4 | -1.5144400 | 0.0460586 | NM_021422 |
| 91 | *Cdc42* | cell division cycle 42 homolog (S. cerevisiae) | -1.5989900 | 0.0462502 | NM_009861 |
| 92 | *Fech* | ferrochelatase | -1.5860000 | 0.0469435 | NM_007998 |
| 93 | *Epha7* | Eph receptor A7 | -1.5415100 | 0.0470891 | NM_001122889 /// NM_010141 |
| 94 | *Glra2* | glycine receptor, alpha 2 subunit | -2.9472500 | 0.0477045 | NM_183427 |
| 95 | *2610005L07Rik /// 6820431F20Rik* | cadherin 11 pseudogene /// cadherin 11 pseudogene | -1.5551400 | 0.0482263 | NR_028428 /// NR_030708 /// XM_001481304 /// XR_001562 |
| 96 | *Pnma1* | paraneoplastic antigen MA1 | -2.4966100 | 0.0484445 | NM_027438 |
| 97 | *Ttc4* | tetratricopeptide repeat domain 4 | -1.5035100 | 0.0485941 | NM_001172073 /// NM_028209 |
| 98 | *Akap5* | A kinase (PRKA) anchor protein 5 | -1.5375700 | 0.0492427 | NM_001101471 /// XM_138063 /// XM_915706 |
| 99 | *Tmtc1* | transmembrane and tetratricopeptide repeat containing 1 | -1.9387500 | 0.0494799 | NM_198967 |
| 100 | *Cspp1* | centrosome and spindle pole associated protein 1 | -1.7878200 | 0.0496262 | NM_026493 |
| 101 | *Gabrg2* | gamma-aminobutyric acid (GABA) A receptor, subunit gamma 2 | -2.1043100 | 0.0496462 | NM_008073 /// NM_177408 |
| 102 | *Ing3* | inhibitor of growth family, member 3 | -1.5383100 | 0.0498631 | NM_023626 |
| 103 | *Lztfl1* | leucine zipper transcription factor-like 1 | -1.5735900 | 0.0499748 | NM_033322 |

**Legend:** List of genes displaying a significant down-regulation at E18.5 (> 1.5 fold decrease, *P* < 0.05) in FACS-isolated *GAD65*-GFP+ cortical interneurons in fluoxetine compared to control condition. Genes are ranked from lowest to highest *P* value scores. Genes labelled in red correspond to genes also significantly dysregulated in the SERT-ko condition.

**Supplementary Table 4:** Genes up-regulated in *GAD65*-GFP+ interneurons during cortical invasion at E18.5 in SERT-ko compared to control condition.

|  | **Gene Symbol** | **Gene Title** | **Fold-Change** | **p-value** | **RefSeq Transcript ID** |
| --- | --- | --- | --- | --- | --- |
| 1 | *Il17rd* | interleukin 17 receptor D | 1.68905 | 0.00159414 | NM_134437 |
| 2 | *Zic1* | zinc finger protein of the cerebellum 1 | 2.7562 | 0.00235592 | NM_009573 |
| 3 | *Zic4* | zinc finger protein of the cerebellum 4 | 1.85266 | 0.00254219 | NM_009576 |
| 4 | *Zic3* | zinc finger protein of the cerebellum 3 | 3.71835 | 0.00283348 | NM_009575 |
| 5 | *Islr2* | immunoglobulin superfamily containing leucine-rich repeat 2 | 2.19953 | 0.0028739 | NM_001161535 /// NM_001161536 /// NM_001161537 /// NM_001161538 /// NM_001161539 |
| 6 | *Plch1* | phospholipase C, eta 1 | 1.69006 | 0.00412634 | NM_001177732 /// NM_001177733 /// NM_183191 |
| 7 | *Foxp2* | forkhead box P2 | 1.91034 | 0.00424962 | NM_053242 /// NM_212435 |
| 8 | *Ddx3y* | DEAD (Asp-Glu-Ala-Asp) box polypeptide 3, Y-linked | 1.81777 | 0.00436705 | NM_012008 |
| 9 | *Wars* | tryptophanyl-tRNA synthetase | 1.62873 | 0.00503885 | NM_001164314 /// NM_001164488 /// NM_011710 |
| 10 | *Six3* | sine oculis-related homeobox 3 homolog (Drosophila) | 1.97195 | 0.00710288 | NM_011381 |
| 11 | *Slc18a2* | solute carrier family 18 (vesicular monoamine), member 2 | 5.10009 | 0.00741678 | NM_172523 |
| 12 | *Ntn1* | netrin 1 | 1.53575 | 0.00836813 | NM_008744 |
| 13 | *Cdkn1b* | cyclin-dependent kinase inhibitor 1B | 1.62339 | 0.00958076 | NM_009875 |
| 14 | *Zfhx3* | zinc finger homeobox 3 | 2.21055 | 0.0102641 | NM_007496 |
| 15 | *Otx2* | orthodenticle homolog 2 (Drosophila) | 2.47743 | 0.0113891 | NM_144841 |
| 16 | *Hjurp* | Holliday junction recognition protein | 1.98626 | 0.0118755 | NM_198652 /// XM_001471840 |
| 17 | *Dcbld1* | discoidin, CUB and LCCL domain containing 1 | 1.61107 | 0.0121674 | NM_025705 |
| 18 | *Zic2* | zinc finger protein of the cerebellum 2 | 1.66806 | 0.0122382 | NM_009574 |
| 19 | *Zfp68* | zinc finger protein 68 | 1.51407 | 0.0141175 | NM_001044747 /// NM_001163797 /// NM_013844 |
| 20 | *Apc2* | adenomatosis polyposis coli 2 | 1.50247 | 0.0141256 | NM_011789 |
| 21 | *Asb4* | ankyrin repeat and SOCS box-containing 4 | 1.95725 | 0.0154462 | NM_023048 |
| 22 | *Fzd3* | frizzled homolog 3 (Drosophila) | 1.61281 | 0.0162202 | NM_021458 |
| 23 | *Pvrl3* | poliovirus receptor-related 3 | 2.83134 | 0.0165476 | NM_021495 /// NM_021496 /// NM_021497 |
| 24 | *Olfm4* | olfactomedin 4 | 1.58271 | 0.0173781 | NM_001030294 |
| 25 | *Nup160* | nucleoporin 160 | 1.50725 | 0.0189094 | NM_021512 |
| 26 | *Slit2* | slit homolog 2 (Drosophila) | 1.50418 | 0.0210535 | NM_178804 |
| 27 | *Kdm5d* | lysine (K)-specific demethylase 5D | 2.15177 | 0.0212812 | NM_011419 |
| 28 | *Sin3a* | transcriptional regulator, SIN3A (yeast) | 1.66765 | 0.0241933 | NM_001110350 /// NM_001110351 /// NM_011378 |
| 29 | *Cotl1* | coactosin-like 1 (Dictyostelium) | 1.52477 | 0.0250333 | NM_028071 |
| 30 | *Marcks* | myristoylated alanine rich protein kinase C substrate | 1.53928 | 0.0282143 | NM_008538 |
| 31 | *Plcxd2* | phosphatidylinositol-specific phospholipase C, X domain containing 2 | 1.8163 | 0.0292197 | NM_001134480 /// XM_001481102 /// XM_489641 |
| 32 | *Ak2 /// LOC100047005* | adenylate kinase 2 /// similar to adenylate kinase 2 | 1.50933 | 0.0293574 | NM_001033966 /// NM_016895 /// XM_001477790 |
| 33 | *Asb4* | ankyrin repeat and SOCS box-containing 4 | 1.81905 | 0.0303597 | NM_023048 |
| 34 | *Aldh1a3* | aldehyde dehydrogenase family 1, subfamily A3 | 3.23323 | 0.0313154 | NM_053080 |
| 35 | *Uty* | ubiquitously transcribed tetratricopeptide repeat gene, Y chromosome | 2.02584 | 0.0319137 | NM_009484 |
| 36 | *Nnt* | nicotinamide nucleotide transhydrogenase | 1.60555 | 0.0325786 | NM_008710 /// NR_003544 |
| 37 | *Ebf1* | early B-cell factor 1 | 2.55853 | 0.0349123 | NM_007897 |
| 38 | *Plod2* | procollagen lysine, 2-oxoglutarate 5-dioxygenase 2 | 1.63638 | 0.0369665 | NM_001142916 /// NM_011961 |
| 39 | *Ralgapa1* | Ral GTPase activating protein, alpha subunit 1 | 1.96518 | 0.0372294 | NM_001003719 /// NM_001112714 /// NM_019994 |
| 40 | *Gprc5b* | G protein-coupled receptor, family C, group 5, member B | 1.8002 | 0.038074 | NM_022420 |
| 41 | *Peli2* | pellino 2 | 1.54592 | 0.03867 | NM_033602 |
| 42 | *Samd4* | sterile alpha motif domain containing 4 | 1.51026 | 0.0414562 | NM_001037221 /// NM_001163433 /// NM_028966 |
| 43 | *Ogt* | O-linked N-acetylglucosamine (GlcNAc) transferase | 1.78004 | 0.0444037 | NM_139144 |
| 44 | *Zfhx3* | zinc finger homeobox 3 | 2.50408 | 0.0472993 | NM_007496 |
| 45 | *Syne2* | synaptic nuclear envelope 2 | 1.53794 | 0.0481647 | NM_001005510 /// XM_001001846 /// XM_917083 |

**Legend:** List of genes displaying a significant up-regulation at E18.5 (> 1.5 fold increase, *P* < 0.05) in FACS-isolated *GAD65*-GFP+ cortical interneurons in SERT-ko compared to control condition. Genes are ranked from lowest to highest *P* value scores. Genes labelled in red correspond to genes also significantly dysregulated in the fluoxetine condition.

**Supplementary Table 5:** Genes down-regulated in *GAD65*-GFP+ interneurons during cortical invasion at E18.5 in fluoxetine compared to SERT-ko condition.

|  | **Gene Symbol** | **Gene Title** | **Fold-Change** | **p-value** | **RefSeq Transcript ID** |
| --- | --- | --- | --- | --- | --- |
| 1 | *Hmgcs1* | 3-hydroxy-3-methylglutaryl-Coenzyme A synthase 1 | -1.56099 | 0.0104511 | NM_145942 |
| 2 | *Ddhd2* | DDHD domain containing 2 | -1.52682 | 0.0123269 | NM_028102 /// XM_356065 /// XM_911470 /// XM_983745 |
| 3 | *Ntrk2* | neurotrophic tyrosine kinase, receptor, type 2 | -1.50013 | 0.0166786 | NM_001025074 /// NM_008745 |
| 4 | *Tusc1* | tumor suppressor candidate 1 | -1.52695 | 0.0193095 | NM_026954 |
| 5 | *Hpca* | hippocalcin | -1.6287 | 0.0231832 | NM_001130419 /// NM_010471 |
| 6 | *Dio2* | deiodinase, iodothyronine, type II | -1.50427 | 0.0245846 | NM_010050 |
| 7 | *Trp53rk* | transformation related protein 53 regulating kinase | -1.50512 | 0.0271708 | NM_023815 |
| 8 | *Hpvc-ps* | Human papillomavirus 18 E5 central sequence motif, pseudogene | -1.55692 | 0.030357 | NM_008283 |
| 9 | *Ppp1cb* | protein phosphatase 1, catalytic subunit, beta isoform | -1.59577 | 0.036342 | NM_172707 |
| 10 | *Meg3* | maternally expressed 3 | -1.55223 | 0.038927 | NR_003633 /// NR_027651 /// NR_027652 /// XR_035480 /// XR_035483 /// XR_035484 |
| 11 | *Hivep3 /// LOC100045240* | human immunodeficiency virus type I enhancer binding protein 3 /// hypothetical | -1.52715 | 0.0405468 | NM_010657 /// XM_001473915 |
| 12 | *Myt1l* | myelin transcription factor 1-like | -1.50634 | 0.0458587 | NM_001093775 /// NM_001093776 /// NM_001093778 /// NM_008666 /// XM_001480703 |
| 13 | *Cacna1b* | calcium channel, voltage-dependent, N type, alpha 1B subunit | -1.53419 | 0.0483889 | NM_001042528 /// NM_007579 |

**Legend:** List of genes displaying a significant down-regulation at E18.5 (> 1.5 fold decrease, *P* < 0.05) in FACS-isolated *GAD65*-GFP+ cortical interneurons in SERT-ko compared to control condition. Genes are ranked from lowest to highest *P* value scores. Genes labelled in red correspond to genes also significantly dysregulated in the fluoxetine condition.

**Supplementary Movies**

**Supplementary Movie 1.** Time-lapse sequence showing GAD65-GFP+ interneurons (INs) migrating in the marginal zone (MZ), cortical plate (CP) and intermediate zone (IZ) between E17.5 and E18.5. End image of the movie depicts examples of migratory tracks of GAD65-GFP+ INs. Scale bar: 100 µm.

**Supplementary Movie 2.** Time-lapse sequence showing GAD65-GFP+ interneurons (INs) migrating in the marginal zone (MZ), cortical plate (CP) and intermediate zone (IZ) between E17.5 and E18.5. End image of the movie depicts examples of migratory tracks of GAD65-GFP+ INs. Scale bar: 25 µm.

**Supplementary Movie 3.** Time-lapse sequence showing GAD65-GFP+ interneurons (INs) exposed *in vivo* to prenatal fluoxetine from E14.5 to E17.5 and migrating in the marginal zone (MZ), cortical plate (CP) and intermediate zone (IZ) between E17.5 and E18.5. End image of the movie depicts examples of migratory tracks of GAD65-GFP+ INs. Scale bar: 25 µm.

**Supplementary Movie 4.** Time-lapse sequence showing SERT-ko; GAD65-GFP+ interneurons (INs) migrating in the marginal zone (MZ), cortical plate (CP) and intermediate zone (IZ) between E17.5 and E18.5. End image of the movie depicts examples of migratory tracks of SERT-ko; GAD65-GFP+ INs. Scale bar: 25 µm.
